# Incidence and survival of oesophageal and gastric cancer in England between 1998 and 2007, a population-based study

**DOI:** 10.1186/1471-2407-12-11

**Published:** 2012-01-12

**Authors:** Victoria H Coupland, William Allum, Jane M Blazeby, Michael A Mendall, Richard H Hardwick, Karen M Linklater, Henrik Møller, Elizabeth A Davies

**Affiliations:** 1 King's College London, Thames Cancer Registry, 1st Floor Capital House, 42, Weston Street, London SE1 3QD, UK; 2 Royal Marsden Hospital, London, UK; 3 University of Bristol, Bristol, UK; 4 Mayday Hospital, Croydon, UK; 5 Addenbrooke's Hospital, Cambridge, UK; 6 University Hospitals Bristol NHS Foundation Trust, Bristol, UK

## Abstract

**Background:**

Major changes in the incidence of oesophageal and gastric cancers have been reported internationally. This study describes recent trends in incidence and survival of subgroups of oesophageal and gastric cancer in England between 1998 and 2007 and considers the implications for cancer services and policy.

**Methods:**

Data on 133,804 English patients diagnosed with oesophageal and gastric cancer between 1998 and 2007 were extracted from the National Cancer Data Repository. Using information on anatomical site and tumour morphology, data were divided into six groups; upper and middle oesophagus, lower oesophagus, oesophagus with an unspecified anatomical site, cardia, non-cardia stomach, and stomach with an unspecified anatomical site. Age-standardised incidence rates (per 100,000 European standard population) were calculated for each group by year of diagnosis and by socioeconomic deprivation. Survival was estimated using the Kaplan-Meier method.

**Results:**

The majority of oesophageal cancers were in the lower third of the oesophagus (58%). Stomach with an unspecified anatomical site was the largest gastric cancer group (53%). The incidence of lower oesophageal cancer increased between 1998 and 2002 and remained stable thereafter. The incidence of cancer of the cardia, non-cardia stomach, and stomach with an unspecified anatomical site declined over the 10 year period. Both lower oesophageal and cardia cancers had a much higher incidence in males compared with females (M:F 4:1). The incidence was also higher in the most deprived quintiles for all six cancer groups. Survival was poor in all sub-groups with 1 year survival ranging from 14.8-40.8% and 5 year survival ranging from 3.7-15.6%.

**Conclusions:**

An increased focus on prevention and early diagnosis, especially in deprived areas and in males, is required to improve outcomes for these cancers. Improved recording of tumour site, stage and morphology and the evaluation of focused early diagnosis programmes are also needed. The poor long-term survival reinforces the need for early detection and multidisciplinary care.

## Background

In 2007, oesophageal cancer was the seventh most common cancer in England in males and the fourteenth in females, with age-standardised incidence rates (per 100,000 European standard population) of around 14.0 and 5.3, respectively [1]. Despite the declining trend in the incidence of stomach cancer in developed countries over the last century it was still the eighth most common cancer in England in males and the sixteenth in females in 2007 [1]. The population mortality rates from oesophageal and stomach cancer closely reflect the population incidence because of the poor prognosis of these cancers.

Over the last 30 years the incidence of adenocarcinoma of the oesophagus (primarily located in the lower third) and cancers of the gastric cardia have been increasing in many developed countries, particularly in males [2-12]. A US study in 1991 found that the annual increase of oesophageal adenocarcinoma in males was greater than any other malignancy in that population [3]. The fact that the increase in incidence has been predominately in males suggests that the rising trends in these cancers cannot fully be explained by changing classifications of the diseases or improvements in diagnosis [7,8]. Both adenocarcinoma of the oesophagus and cancer of the gastric cardia are typically characterised by high male to female ratios, with the incidence in males often being reported as up to four times higher than that of females [3,5,6]. Internationally, the highest incidence of oesophageal adenocarcinoma has been reported in the United Kingdom [13].

Recent national studies have described the incidence and survival of oesophageal and stomach cancer [14-16], but have not explored specific subgroups of these cancers. This study aims to describe the incidence and survival of patients with oesophagogastric cancers in England using a recent national cohort of patients diagnosed between 1998 and 2007.

## Methods

Data on 133,804 patients (85,361 males; 48,443 females) diagnosed with oesophageal and gastric (OG) cancer in England between 1998 and 2007 were extracted from the National Cancer Data Repository (NCDR). The NCDR contains information collected by the eight English cancer registries on all cancer patients diagnosed in their respective catchment areas. These data are validated and quality assured in each registry before being combined into the English dataset.

These data are divided into six groups: 1) upper and middle oesophagus, 2) lower oesophagus, 3) oesophagus with unspecified anatomical site (from now on referred to as oesophagus not otherwise specified (NOS)), 4) cardia, 5) non-cardia stomach, and 6) stomach with unspecified anatomical site (from now on referred to as stomach not otherwise specified (NOS)). These groups were defined primarily on the basis of ICD10 (International Classification of Diseases version 10) codes (see Table 1). However, as the majority of oesophageal adenocarcinoma is found in the lower third of the oesophagus [7,17] while squamous cell carcinoma is more commonly found in the upper and middle oesophagus [17], some patients with an oesophageal NOS cancer were assigned to the upper and middle or lower oesophageal subgroups based on their histological diagnosis.

**Table 1:** Oesophago-gastric cancer group definitions

Oesophago-gastric cancer groups	International Classification of Diseases version 10 (ICD10) and International Classification of Diseases for Oncology (ICDO2) codes
Upper and middle oesophagus	C15.0-C15.1, C15.3-C15.4
	including C15.8-C15.9 with a morphology code 8050-8083 (Squamous cell carcinomas)

Lower oesophagus	C15.2, C15.5
	including C15.8-C15.9 with a morphology code 8140-8576 (Adenocarcinomas)

Oesophagus not otherwise specified	C15.8-C15.9
	excluding C15.8-C15.9 with a morphology code 8050-8083 (Squamous cell carcinomas) or 8140-8576 (Adenocarcinomas)

Cardia	C16.0

Non-cardia	C16.1-C16.6

Stomach not otherwise specified	C16.8-C16.9

For each cancer group age specific incidence rates were calculated in 5 year age groups ranging from 0-4 through to 85 and over for males and females. Age-standardised incidence rates (per 100,000 European standard population, ASR(E)) were calculated for each of the six cancer groups by year of diagnosis and sex. ASR(E) were also calculated by socioeconomic deprivation quintile for patients diagnosed between 2003 and 2007. Postcodes were used to assign each patient to a lower super output area (LSOA) of residence--(LSOA are areas including around 1,500 people). Deprivation quintiles were assigned based on these LSOAs using the income domain of the Indices of Deprivation 2007 [18]. Male to female incidence rate ratios were calculated for each group and year of diagnosis.

Survival was estimated using the Kaplan-Meier method for each of the six patient groups. Survival was based on patients diagnosed between 1998 and 2007 and followed up until the end of 2007. 4,990 (3.7%) of the 133,804 patients were death certificate only registrations and were excluded from the survival analysis as they had no relevant date of diagnosis, leaving 128,814 patients. The total person time of follow-up was 142,187 years.

A sensitivity analysis was also carried out. Oesophageal cancer patients were assigned to one of three morphological groups; adenocarcinoma, squamous cell carcinoma and "other and unspecified types." The incidence and survival analyses were repeated for these three groups.

Cancer registries in England have approval from the National Information Governance Board to carry out surveillance using the data they collect on all cancer patients under section 251 of the NHS Act 2006. Therefore separate ethical approval was not required for this study.

## Results

### Upper and middle oesophageal cancer

**Figure 1:**
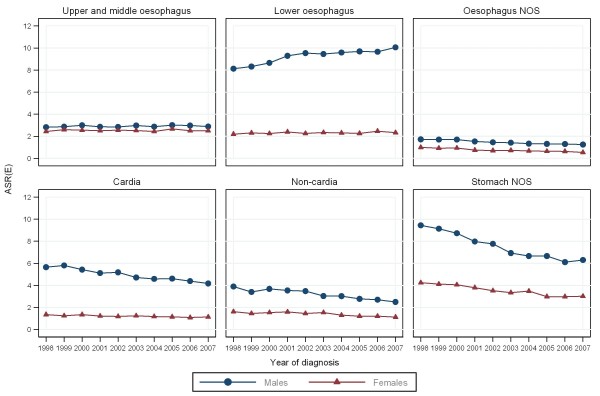
**Age-standardised incidence rates (per 100,000 European standard population) for patients diagnosed with oesophago-gastric cancers in England between 1998 and 2007**.

**Figure 2:**
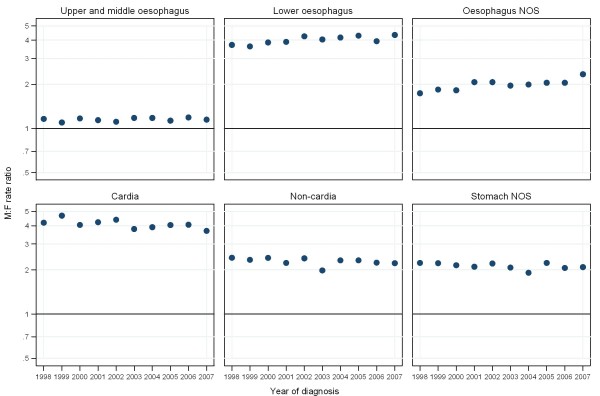
**Male to female incidence rate ratios for patients diagnosed with oesophago-gastric cancers in England between 1998 and 2007**.

**Figure 3:**
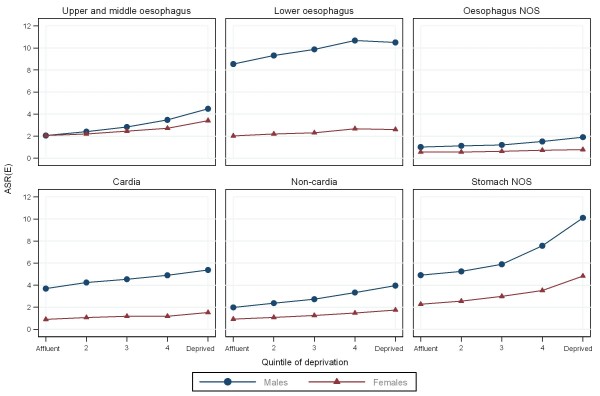
**Age-standardised incidence rates (per 100,000 European standard population) for patients diagnosed with oesophago-gastric cancers in England between 2003 and 2007, by deprivation quintile**.

**Figure 4:**
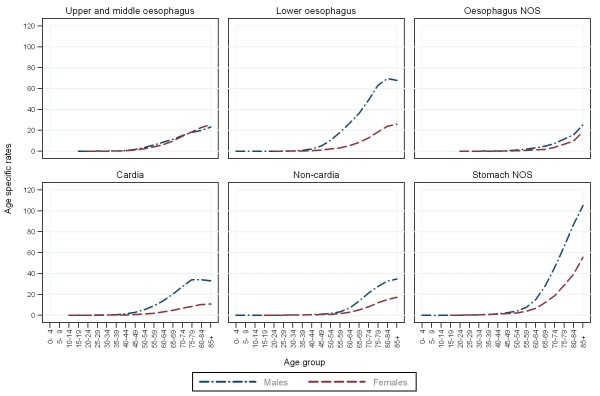
**Age specific incidence rates for patients diagnosed with oesophago-gastric cancers in England between 1998 and 2007**.

**Figure 5:**
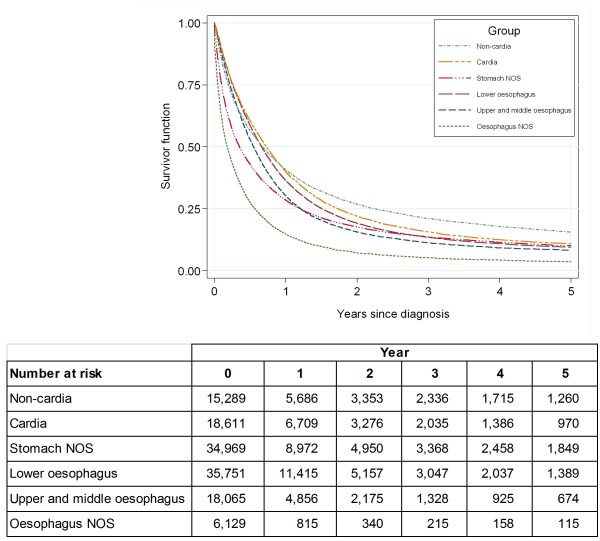
**Kaplan-Meier survival functions for oesophago-gastric cancers diagnosed in England between 1998 and 2007**. Followed up to the end of December 2007.

Over half of upper and middle oesophageal cancers were in females (Table 2). The median age at diagnosis was 73 years. Incidence remained constant over time and was similar in males and females (Figures 1, and 2). Incidence was higher in more deprived areas, especially in males where the ratio between the most deprived (quintile 5 (Q5)) and the most affluent (quintile 1 (Q1)) groups was 2.2:1 males and 1.7:1 females (Figure 3). The age specific incidence rates were similar in males and females (Figure 4). 30.3% [95% confidence interval 29.6-31.0%] of patients survived 1 year and 8.3% [7.8-8.7%] survived five years after diagnosis (Figure 5). The results for the oesophageal squamous cell carcinoma group were similar to the upper and middle oesophageal cancer group.

**Table 2:** Characteristics of patients diagnosed with oesophago-gastric cancers in England between 1998 and 2007

	Upper and middle oesophagus		Lower oesophagus		Oesophagus not otherwise specified		Cardia		Non-cardia		Stomach not otherwise specified		Oesophageal and gastric cancer	
	**18,128**		**35,849**		**7,898**		**18,728**		**15,340**		**37,861**		**133,804**	

Male	8,228	(45.4)	26,495	(73.9)	4,323	(54.7)	14,107	(75.3)	9,531	(62.1)	22,677	(59.9)	85,361	(63.8)

Female	9,900	(54.6)	9,354	(26.1)	3,575	(45.3)	4,621	(24.7)	5,809	(37.9)	15,184	(40.1)	48,443	(36.2)

Death certificate only (DCO)	63	(0.3)	98	(0.3)	1,769	(22.4)	117	(0.6)	51	(0.3)	2,892	(7.6)	4,990	(3.7)

Median age	73		72		78		71		75		76		74	

< 50	706	(3.9)	1,682	(4.7)	180	(2.3)	1,043	(5.6)	595	(3.9)	1,568	(4.1)	5,774	(4.3)

50-54	937	(5.2)	1,986	(5.5)	227	(2.9)	1,025	(5.5)	384	(2.5)	961	(2.5)	5,520	(4.1)

55-59	1,513	(8.3)	3,149	(8.8)	396	(5.0)	1,626	(8.7)	699	(4.6)	1,671	(4.4)	9,054	(6.8)

60-64	1,864	(10.3)	3,980	(11.1)	552	(7.0)	2,124	(11.3)	1,196	(7.8)	2,646	(7.0)	12,362	(9.2)

65-69	2,320	(12.8)	4,775	(13.3)	707	(9.0)	2,680	(14.3)	2,010	(13.1)	4,326	(11.4)	16,818	(12.6)

70-74	2,851	(15.7)	5,687	(15.9)	1,030	(13.0)	3,162	(16.9)	2,735	(17.8)	6,013	(15.9)	21,478	(16.1)

75-79	3,018	(16.6)	6,170	(17.2)	1,444	(18.3)	3,185	(17.0)	3,056	(19.9)	7,293	(19.3)	24,166	(18.1)

80-84	2,496	(13.8)	4,735	(13.2)	1,364	(17.3)	2,218	(11.8)	2,497	(16.3)	6,585	(17.4)	19,895	(14.9)

85+	2,423	(13.4)	3,685	(10.3)	1,998	(25.3)	1,665	(8.9)	2,168	(14.1)	6,798	(18.0)	18,737	(14.0)

**Quintile of deprivation 2007**^†^														

1 = (Affluent)	1,503	(16.1)	3,589	(18.8)	592	(16.6)	1,554	(17.5)	1,058	(15.0)	2,607	(15.3)	10,903	(16.8)

2	1,793	(19.2)	4,133	(21.6)	701	(19.7)	1,855	(20.9)	1,320	(18.7)	3,094	(18.1)	12,896	(19.8)

3	1,980	(21.2)	4,143	(21.7)	741	(20.8)	1,941	(21.8)	1,503	(21.3)	3,409	(20.0)	13,717	(21.1)

4	1,994	(21.4)	4,000	(20.9)	791	(22.2)	1,811	(20.4)	1,625	(23.0)	3,782	(22.2)	14,003	(21.5)

5 = (Deprived)	2,058	(22.1)	3,256	(17.0)	738	(20.7)	1,730	(19.5)	1,561	(22.1)	4,180	(24.5)	13,523	(20.8)

**Year of diagnosis**														

1998	1,702	(9.4)	3,067	(8.6)	929	(11.8)	2,022	(10.8)	1,752	(11.4)	4,446	(11.7)	13,918	(10.4)

1999	1,754	(9.7)	3,191	(8.9)	905	(11.5)	2,020	(10.8)	1,546	(10.1)	4,301	(11.4)	13,717	(10.3)

2000	1,805	(10.0)	3,292	(9.2)	923	(11.7)	2,014	(10.8)	1,669	(10.9)	4,261	(11.3)	13,964	(10.4)

2001	1,751	(9.7)	3,574	(10.0)	810	(10.3)	1,854	(9.9)	1,679	(10.9)	3,959	(10.5)	13,627	(10.2)

2002	1,788	(9.9)	3,604	(10.1)	768	(9.7)	1,927	(10.3)	1,627	(10.6)	3,822	(10.1)	13,536	(10.1)

2003	1,861	(10.3)	3,678	(10.3)	745	(9.4)	1,827	(9.8)	1,565	(10.2)	3,538	(9.3)	13,214	(9.9)

2004	1,793	(9.9)	3,732	(10.4)	717	(9.1)	1,777	(9.5)	1,472	(9.6)	3,514	(9.3)	13,005	(9.7)

2005	1,920	(10.6)	3,813	(10.6)	730	(9.2)	1,820	(9.7)	1,375	(9.0)	3,390	(9.0)	13,048	(9.8)

2006	1,871	(10.3)	3,887	(10.8)	722	(9.1)	1,740	(9.3)	1,369	(8.9)	3,264	(8.6)	12,853	(9.6)

2007	1,883	(10.4)	4,011	(11.2)	649	(8.2)	1,727	(9.2)	1,286	(8.4)	3,366	(8.9)	12,922	(9.7)

^†^Based on patients diagnosed between 2003 and 2007.

### Lower oesophageal cancer

The majority of oesophageal cancers were located in the lower oesophagus, and almost three quarters were in males (Table 2). The median age at diagnosis was 72 and the number of cases increased over the period. Lower oesophageal cancer in males had the highest incidence of all the oesophageal groups. The incidence rose from 8.1 per 100,000 in 1998 to 10.1 in 2007 and was relatively stable after 2002 (Figure 1). The difference in incidence between males and females was most evident in this subgroup (M:F 4:1) and incidence was higher in more deprived areas (Q5:Q1 1.2:1 males; 1.3:1 females) (Figures 2, and 3). 36.4% [35.9-36.8%] of patients survived 1 year and 9.4% [9.1-9.8%] survived 5 years after diagnosis (Figure 5). The results for the oesophageal adenocarcinoma group were similar to the lower oesophageal cancer group.

### Oesophageal NOS

A small proportion of oesophageal cancer patients had NOS disease and the median age at diagnosis was 78 (Table 2). The incidence decreased over the period, and was higher in males than females, and in more deprived areas (Figures 1, 2, and 3). 14.8% [13.9-15.7%] of patients survived 1 year and 3.7% [3.2-4.3%] survived 5 years after diagnosis (Figure 5). The results for the oesophageal other and unspecified morphology group were similar to the oesophageal NOS cancer group.

### Cardia

Over three quarters of cardia cancers were in males (Table 2). The median age at diagnosis was 71. Incidence declined slightly over the period falling from 5.7 to 4.2 per 100,000 in males and from 1.4 to 1.1 in females (Figure 1). Like lower oesophageal cancer the incidence of cardia cancer was much higher in males than females (M:F 4:1) and was higher in the most deprived quintiles (Q5:Q1 1.5:1 males; 1.7:1 females) (Figures 2, and 3). 40.0% [39.3-40.7%] of patients survived 1 year and 10.9% [10.4-11.4%] survived 5 years after diagnosis (Figure 5).

### Non-cardia

Sixty-two per cent of non-cardia stomach cancers were in males (Table 2). The median age at diagnosis was 75 and the annual number of cases declined over time. Incidence also declined, was twice as high in males, and was higher in more deprived areas (Q5:Q1 2.0:1 males; 1.9:1 females) (Figures 1, 2 and 3). 40.8% [40.0-41.6%] of patients survived 1 year and 15.6% [15.0-16.3%] survived 5 years after diagnosis (Figure 5).

### Stomach NOS

Over half of stomach cancers were NOS (Table 2). The median age at diagnosis was 76. The incidence fell from 9.4 per 100,000 in 1998 to 6.3 in 2007 in males and from 4.2 to 3.0 in females (Figure 1). Incidence was higher in males (M:F 2:1), in the more deprived groups (Q5:Q1 2.1:1 in both sexes), and was particularly high in the oldest age groups (Figure 2, 3, and 4). 28.5% [28.0-29.0%] of patients survived 1 year and 10.1% [9.8-10.5%] survived 5 years after diagnosis (Figure 5).

## Discussion

This study investigated the incidence and survival of oesophageal and gastric cancers in England using data on 133,804 patients diagnosed between 1998 and 2007. The incidence of lower oesophageal cancer increased until 2002 then remained relatively stable, whereas the incidence of cancers of the cardia, non-cardia, and stomach NOS declined over this period. The incidence was higher in males compared with females for both oesophageal and gastric cancer. This was most evident in lower oesophageal and cardia cancers where the incidence was around four times higher in males. In general the incidence rates of all oesophageal and gastric cancers were higher in the more deprived areas. Overall survival was poor in all sub-groups with 1 year survival ranging from 14.8 to 40.8% and 5 year survival ranging from 3.7 to 15.6%.

### Strengths and limitations

This national study included a large number of patients diagnosed with oesophageal or stomach cancer over a 10 year period. It was therefore possible to investigate differences in incidence by six cancer subgroups rather than only the traditional groups of oesophageal (C15) and stomach (C16) cancer, which obscure the unique features of the lower oesophageal and cardia tumours. It is also strengthened by available socioeconomic and survival data.

One limitation of the dataset was the relatively large proportion of patients with an unspecified anatomical subsite, particularly for gastric cancers where over half (52.6%) fell into this group. This meant that these patients could not be assigned to either the cardia or non-cardia subgroup. Defining the oesophageal cancer groups using both the anatomical site and tumour morphology led to a smaller proportion of patients in the not otherwise specified group (12.8%) compared to the groups defined and used for the sensitivity analysis based on morphology alone (17.5%). This sensitivity analysis demonstrated similar patterns in incidence and survival between the oesophageal squamous cell carcinoma group and the upper and middle oesophageal group, the oesophageal adenocarcinoma group and the lower oesophageal group, and the "other and unspecified" and the oesophageal not otherwise specified group.

Finally, another limitation was that 4,990 patients (4%) had to be excluded from the survival analysis as their registrations were based only on data from the death certificate.

### Comparison to previous studies

In most developed countries the incidence of oesophageal squamous cell carcinoma, which is more commonly found in the upper and middle oesophagus [17], has remained constant or declined over the last 30 years [11] whilst the incidence of oesophageal adenocarcinoma, primarily found in the lower third of the oesophagus [7,17] has increased, particularly in males [2-12]. In Sweden the increase in oesophageal adenocarcinoma incidence peaked in the mid 2000's and then remained stable [19]. Our study found similar results with stable incidence rates of upper and middle oesophageal cancer over the 10 year period and an initial increase in the incidence of lower oesophageal cancer in males which has slowed and remained relatively stable after 2002.

The increase in the incidence of lower oesophageal cancer is mirrored in our study by a decrease in the incidence of cancers of the cardia. Previous studies have noted an increase in both these cancer groups [2-8], although others have found a similar stable or slightly declining trend in the incidence of gastric cardia cancer since the early 1990's [2,12,19]. It is possible that the trends in these two adjacent sites were influenced by changing diagnostic classification. However, after 2002 when the incidence of lower oesophageal cancer remained stable in males the incidence of cardia cancer continued to decline. If changes in diagnostic classification were responsible these trends would be expected to stabilise at a similar time. It is also possible that some of the oesophageal NOS cancers could have been cardia cancers. Reassigning those with a histological diagnosis of adenocarcinoma to the lower oesophageal group may have influenced the trends. A decline in the incidence of non-cardia gastric cancer in more developed countries has been seen in the past century [8,20-22]. Our study confirms this continued decline for both non-cardia stomach and stomach NOS cancers.

The declining incidence of gastric cancers in this study and other studies [8,20-22] may be associated with the decreasing prevalence in developed countries of *Helicobacter pylori *infection, a known risk factor for gastric cancer [20]. However, meta-analyses have found that infection with the most common *H pylori *strains (CagA+) may protect against the development of oesophageal adenocarcinoma, possibly because infection causes achlorhydria and so reduces gastric acid reflux, one of the main risk factors associated with the development of oesophageal adenocarcinoma [23,24]. A systematic review did find a lower prevalence of *H pylori *infection in patients with gastro-oesophageal reflux disease (GORD) [25]. Therefore, the declining prevalence of *H pylori *infection could contribute to the increasing incidence of lower oesophageal cancer found here.

The increasing incidence of oesophageal adenocarcinoma found in this study may also be associated with the rising prevalence of obesity in England [26]. Other studies have found that increasing body mass index is associated with an increased risk of oesophageal adenocarcinoma and cardia cancer [27,28].

Consistent with previous studies [3-8,12] lower oesophageal and cardia cancer incidence rates were much higher in males compared with females (M:F 4:1). The reasons for this is not clear, but an abdominal distribution of body fat, which is more common in males, may lead to higher levels of GORD and therefore to an increased risk of developing these cancers [27,29]. Barrett's oesophagus, secondary to chronic GORD, is another risk factor which occurs more commonly in males [30] and has been linked to abdominal obesity [27,31]. Differing patterns of past smoking behaviours in males and females could also partly explain the differing incidence of these cancers. The risk of developing squamous cell carcinoma of the oesophagus declines steadily following smoking cessation, although the risk of both oesophageal adenocarcinoma and cardia cancer does not decline until 30 years after cessation [32].

The finding that lower oesophageal and cardia cancer have a higher incidence in the more socioeconomically deprived groups contradicts other studies which have found no association [9]. Squamous cell carcinoma [9] and gastric cancer [20] have been associated with deprivation in previous studies, which our findings support. The known lifestyle risk factors already discussed are likely to be more common in those living in deprived areas and so explain the higher incidence found in our study.

### Implications for policy and practice

The poor prognosis of both oesophageal and gastric cancer highlights the need to concentrate efforts on primary prevention. Smoking and high alcohol consumption are risk factors for gastric cancer and squamous cell carcinoma of the oesophagus [33-35]. Smoking is also a risk factor for oesophageal adenocarcinoma, but the role of alcohol consumption is less certain [34]. Public health initiatives aimed at reducing smoking and encouraging sensible alcohol consumption would help reduce the incidence of these cancers. A systematic review found that reducing weight may improve symptoms of GORD although not all studies have found this association [27]. Other public health initiatives aimed at reducing obesity therefore may help to decrease the prevalence of chronic GORD which is one of the main risk factors for developing oesophageal adenocarcinoma.

The particularly high incidence of lower oesophageal and cardia tumours in males may have implications for earlier diagnosis. Current guidelines for referral and investigation of upper gastrointestinal symptoms do not specify this increased risk in males, but advise a similar threshold for males and females [36]. Raising awareness in primary care of the differing incidence should be considered, and a lower threshold for referral in males investigated. The poor prognosis of all patients suggests that evaluation of a national programme of earlier investigation of non-specific UGI symptoms may be warranted, and new tools such as the cytosponge for identifying Barretts' epithelium may have a role to play in the future [33,37].

Since oesophageal and gastric tumours are relatively uncommon and difficult to diagnose population-wide screening is unlikely to be cost effective. Efforts to identify high risk groups such as those with regular chronic reflux (oesophageal cancer) could perhaps be considered in developing focused screening efforts in the future, but evidence on the effectiveness of screening these groups will be needed. At present endoscopic screening is not considered feasible [33]. However, an American study in 2010 did suggest that the incidence in White males over 60 with weekly GORD or over 55 with daily GORD was high enough to investigate the effectiveness of screening in these groups [38].

The poor prognosis of these cancers also suggests the need for greater focus on earlier diagnosis. Raising public awareness and knowledge of symptoms, particularly in more deprived areas and in males, will be important. A recent study found that 21% of oesophageal and 32% of stomach cancers were diagnosed as a result of emergency admissions and that emergency admissions were associated with poorer one-year survival [39]. Therefore, greater awareness of these cancers and improved knowledge of symptoms could help to identify earlier stage tumours and consequently improve the prognosis of these cancers.

### Unanswered questions and future research

These data highlight the need for further aetiological research to understand the links between sex and oesophageal and gastric cancers. Of particular importance is to fully understand why the incidence rates of lower oesophageal and cardia cancer are so much higher in males compared with females, why the incidence of these cancers are higher in the UK compared with other developed countries and why the incidence has increased over time. This study lacked information on several factors that may affect the incidence of these cancers such as smoking, alcohol, obesity, GORD and *H pylori *infection which, if available, could have helped in the interpretation of the analysis. Future work could record how these factors vary within populations in England. An earlier English study found significantly higher postoperative mortality following operations for oesophageal cancer in patients living in more deprived areas [40]. Future studies could also investigate differences in survival for the six subgroups in this study taking into account other factors that influence survival such as age, socioeconomic deprivation and stage of disease.

Finally, these data also show that better classification of gastric tumours by site is needed to understand outcomes. It is important that both the cancer site and the morphology of these cancers are identified and recorded in clinical practice where possible. This information needs to be passed to the cancer registries to ensure that further studies can investigate these groups with more accuracy.

## Conclusion

In England, the incidence of lower oesophageal cancer in males increased until 2002 then remained relatively stable, whereas the incidence of cardia and non-cardia gastric cancers declined between 1998 and 2007. Cancers of the lower oesophagus and cardia were more likely to occur in males than females and the incidence for all oesophageal and gastric cancer subgroups was higher in more deprived areas. The prognosis for these cancers remains very poor. An increased focus on prevention and early diagnosis, especially in deprived areas and in males, is required to improve outcomes for oesophageal and gastric cancers. Improved recording of tumour site, stage and morphology and the evaluation of focused early diagnosis programmes are also needed. The poor survival reinforces the need for early detection and multidisciplinary care.

## Competing interests

The authors declare that they have no competing interests.

## Authors' contributions

This study was designed by VHC and EAD. KML prepared and managed the dataset and VHC analysed the data. VHC drafted the paper and EAD, HM, KML, WA, JB, MM and RHH reviewed and revised the drafts and provided interpretation of results. All authors have approved the final version of the manuscript.

## Pre-publication history

The pre-publication history for this paper can be accessed here:

http://www.biomedcentral.com/1471-2407/12/11/prepub
